# A year of great achievements

**DOI:** 10.20945/2359-3997000000423

**Published:** 2021-11-24

**Affiliations:** 1 Faculdade de Medicina da Universidade de São Paulo Hospital das Clínicas São Paulo SP Brasil Editor-Chefe da Archives of Endocrinology and Metabolism, Chefe da Unidade de Neuroendocrinologia, Serviço de Endocrinologia e Metabologia, Hospital das Clínicas, Faculdade de Medicina da Universidade de São Paulo (HCFMUSP), São Paulo, SP, Brasil

This is the last issue of the 7th year of The Archives of Endocrinology and Metabolism, formerly *Arquivos Brasileiros de Endocrinologia e Metabologia*, first published 70 years ago. That's also my seventh year as Editor-in-Chief. During this period, the AE&M, official scientific journal of The Brazilian Society of Endocrinology and Metabolism (SBEM), increased its impact factor from 0.6 to 2.309 (1), even considering the COVID-19 pandemics which strongly affected our regular life.

The number of submitted papers from outside Brazil also increased. In fact, in 2015 the Journal received 165 Brazilian manuscripts and 191 from other countries (46.3% *vs.* 53,7%). In 2020 the numbers went to: 190 (33%) *vs.* 387 (67%). Also, the amount of countries increased significantly encompassing all world continents. This improvement could not be achieved without the collaboration of our Associated Editors, Reviewers from Brazil and abroad, editorial assistants, and the support of SBEM's board of Directors.

Recently our site was renewed, becoming more attractive and interactive. Therefore, I will begin my eighth year as Editor aiming to continuously improve AE&M and project it more and more in the international scientific scenario. For this purpose, I count on the outstanding collaboration of all people who are hearty involved with their time and expertise with our Journal and ask all the endocrinology community around the world to submit their manuscripts to us.
